# Evaluation of Pre-Treatment Serum Levels of IL-7 and GM-CSF in Colorectal Cancer Patients

**Published:** 2014

**Authors:** Mehdi Taghipour Fard Ardekani, Mahyar Malekzadeh, Seyed Vahid Hosseini, Elahe Bordbar, Mehrnoosh Doroudchi, Abbas Ghaderi

**Affiliations:** 1 *Shiraz Institute for Cancer Research, School of Medicine, Shiraz University of Medical Sciences, Shiraz, Iran.*; 2 *Department of Immunology, School of Medicine, Shiraz University of Medical Sciences, Shiraz, Iran.*; 3 *Colorectal Research Center, Faghihi Hospital, School of Medicine, Shiraz University of Medical Sciences, Shiraz, Iran.*

**Keywords:** Colorectal cancer, serum, GM-CSF, IL-7

## Abstract

Survival of Colorectal cancer (CRC) patients is considerably stage-dependent; therefore, early diagnosis is a pivotal factor in decreasing mortality and morbidity associated with this cancer. GM-CSF and IL-7 are reported to increase in different cancers and we aimed to investigate the pre-treatment serum levels of GM-CSF and IL-7 in Iranian patients with colorectal cancer. 127 patients (68 males and 59 females) entered this study before receiving chemotherapy or radiotherapy. A control group of 50 healthy age/sex matched individuals (27 males and 23 females) were included in the study. The serum levels of GM-CSF and IL-7 were measured using commercial enzyme linked immunosorbent assays. A significantly higher level of GM-CSF was found in the sera of patients with colorectal cancer compared to healthy age/sex matched controls (P=0.013). However, there was no significant difference between the levels of IL-7 in sera of patients and controls. We observed a significant elevation in the level of GM-CSF in poorly differentiated tumors (P=0.024). Also a significant correlation between lymphatic invasion and the level of GM-CSF in sera of CRC patients was detected (P=0.01). We found an increase of the level of IL-7 in four patients presenting moderate stages of tumor concomitant with a decrease of the level of GM-CSF. It can be concluded that the increase of the level of GM-CSF is accompanied by CRC progression in Iranian patients. Potential therapeutic effect of IL-7 in this disease, however, needs further investigations.

Annually one million new cases of colorectal cancer (CRC) are diagnosed and half a million death occurs due to this cancer (1). It represents the third and second most common cancer in men and women respectively, (10% and 9.4% of all cancers, respectively) but its mortality is less in women than in men. The survival rate of CRC is significantly different in early stages versus late stages (1). In fact, staging is the most important factor in evaluating the prognosis of colorectal cancer (2); therefore, early diagnosis and treatment can reduce mortality and morbidity of this cancer (1).

The immune system plays a dual role in the defence and development of many tumors. Interestingly, tumor cells may exploit or produce effectors of the immune system for their own advantage. Colony Stimulating Factors (CSFs) are among the cytokines that are produced by cancer cells as well as the immune system in the course of tumorigenesis (3). Cytokines can also modulate several processes involved in tumor progression and metastasis, for example angiogenesis and the production of metalloproteinases (4).

Granulocyte Macrophage-Colony Stimulating Factor (GM-CSF) is a cytokine that acts as a growth factor for white blood cells, and stimulates bone marrow to produce granulocytes and macrophages (5). GM-CSF is highly efficient in inducing speciﬁc immune responses resulting in tumor destruction (6). On the other hand, the production of GM-CSF correlates with the increase of recurrence rates in head and neck squamous cell carcinoma, possibly due to an inhibition of immune responsiveness (7). GM-CSF exerts an important role in regulation of intestinal immune and inflammatory responses (8). The establishment of normal colon epithelium is under the tight regulation of GM-CSF by controlling apoptosis and proliferation of these cells; therefore, GM-CSF deregulation is suggested to be a part of colon carcinogenesis. Moreover, GM-CSF can decrease apoptosis in colon cancer (9) and has a role in growth and cancer spreading (10).

On the other hand, CRC cells with microinstability (MSI) gene can generate abnormal peptides that stimulate the secretion of cytokines (including GM-CSF), the infiltration of lymphocytes and stimulate the immune system against tumor, which can lead to a better prognosis for the patients (6). An increase in the level of GM-CSF in sera of colorectal cancer patients was shown to correlate with tumor prognosis (5).

Another cytokine which is shown to be produced by epithelial cells, keratinocytes, dendritic cells, hepatocytes, neurons, but not lymphocytes is Interleukin 7 (IL-7) (11). The expression of functional IL-7 receptor (IL-7R) on epithelial tumor cells including a colon cancer cell line was reported (12). IL-7 is a glycoprotein that is normally secreted by stromal cells in the red marrow and thymus and stimulates the proliferation of pre-B and pro-B cells while it supports the maturation of megakaryocytes and stimulates the proliferation of early and mature activated T-cells (13). It is responsible of increasing the production of cytotoxic T cells and T killer cells and induces proinflammatory cytokine secretion and the anti-tumor activity of monocytes and T cells (14). IL-7 also enhances the expression and secretion of IL-3 and GM-CSF in activated human T-cells and downregulates TGF-beta in macro-phages, thereby accelerating anti-tumor immune responses (15).

Experimental immunodeficient tumor-bearing mice have provided the evidence of the anti-tumor properties of recombinant human IL-7 (rhIL-7) on a human colon tumor (16). In addition, vaccination of patients with progressive colon cancer disease with autologous tumor cells transfected with IL-7 and GM-CSF genes could result in regression of tumor in some patients (17). Conversely, IL-7 is reported to be elevated in sera of patients with colorectal cancer at stages III and IV. This notion has resulted in considering IL-7 as a diagnostic or prognostic factor in this cancer (18**-**20).

Increased GM-CSF mRNA, protein and its receptor have been found in many colon cancer cell lines, as well as in surgical specimens (21-22). GM-CSF and Macrophage-Colony Stimulating Factor (M-CSF) were reported to stimulate the metastatic properties of carcinoma cell lines and correlate with tumor prognosis (5, 23-24). There are few reports that have examined GM-CSF in the pathogenesis of colorectal cancer, and the studies on the level of IL-7 in these patients are scarcer.

In the present study we aimed to determine the pre-treatment serum levels of GM-CSF and IL-7 in Iranian patients with CRC and investigate their possible correlation with clinicopathological chara-cteristics of the patients. The potential diagnostic use of circulating GM-CSF and IL-7 and further clinical applications in Iranian patients with colorectal cancer were explored as preliminary data.

## Materials and Methods

**Patients**

This study was approved by the Ethics Committee of Shiraz University of Medical Science (SUMS). The patients were informed about the aim of this study as well as safety and security measures before their consents were obtained. The cases were selected among CRC patients who were referred for surgery to hospitals related to SUMS between November 2009 and September 2010. 127 cases (68 males and 59 females) aged between 13-83 years entered the study. None of the patients had been treated by chemotherapy or radiotherapy before sample collection. The clinicopathological charac-teristics of the patient group is shown in table 1.

**Table 1:** Clinicopathological characteristics of patients

Characteristics	Number (127)	Percentage	Characteristics	Number (127)	Percentage
Tumor type Adenocarcinoma Adenocarcinoma with mucin production other	106 16 5	83.5 12.6 3.9	Lymph node inv.* Is seen Not seen Unknown	38 60 29	29.9 47.3 22.8
Histological grade Well diff. Modorate diff Poorly diff. Unknown	86 17 11 13	67.7 13.4 08.7 10.2	T T1 T2 T3 T4 Unknown	08 25 55 18 21	06.3 19.7 43.3 14.2 16.5
Tumor size Diameter>5cm Diameter<5 cm Unknown	79 19 29	62.2 15 22.8	N N0 N1 N2 Unknown	57 22 16 32	44.9 17.3 12.6 25.2
Tumor side Right colon Left colon Unknown	13 84 30	10.2 66.2 23.6	M M0 M1 Unknown	90 27 10	70.9 21.3 07.9
Perilymphatic inv. Is seen Not seen Unknown	26 98 3	20.5 77.1 2.4	Tumor stage Stage 1 (Low) Stage 2 (Low) Stage 3 (High) Stage 4 (High) Unknown	25 40 23 30 9	19.7 31.5 18.1 23.6 07.1
Perineural inv. Is seen Not seen Unknown	14 110 3	11.0 86.6 2.4	Depth of invasion mucosa and submucosa mascularis propria serosa & subserosa Unknown	07 27 68 25	05.5 21.3 53.5 19.7
Perivascular inv. Is seen Not seen Unknown	18 106 3	14.2 83.4 2.4			

* Inv.= Involvement

The control group was selected from 50 healthy adults aged between 21-83 years (27 males and 23 females) who had no acute or chronic diseases such as autoimmune disease, diabetes mellitus, thyroid disease, recent common cold, hypertension, hyper-lipidemia, ischemic heart disease, cerebrovascular, renal, skin and pulmonary disorders as well as infectious diseases and were not receiving any medications.

The group of 127 CRC patients (68 males and 59 females) and 50 healthy controls (27 males and 23 females) were matched based on age and gender (Table 2). The mean age of the CRC patients was 54.27 ± 15.62 years and the mean age of healthy individuals was 54.38 ± 13.98 years. Similarly, the female/ male ratio in the CRC group was 0.87 and in control group was 0.85 (P= 0.956).

**Samples**

Four ml blood was collected from peripheral veins of patients on the day before surgery. The samples were brought to Shiraz Institute for Cancer Research (ICR) immediately. Samples were centrifuged and sera were preserved at -20˚C till used. On the day of operation, the tissue biopsies were assessed by collaborative pathologist. The pathologist confirmed colon cancer and evaluated invasion of tumors to perineural, perivascular, perilymphatic and local area lymph nodes. Cancer was staged according to tumor-node-metastasis (TNM) by the American Joint Committee on Cancer Classification and stage grouping.

**ELISA assays**

The plasma level of GM-CSF was measured by a commercial enzyme linked immunosorbent assay (ELISA) (eBiosciences, Austria) according to the manufacturer's instructions. The sensitivity of this assay was 0.6 pg/ml and the range of detection was between 7.8-500 pg/ml. The serum level of IL-7 was measured using a commercial ELISA assay (Abcam, UK) according to the manufacturer's instructions. The sensitivity of this assay was less than 3 pg/ml and the range of detection was between 6.25-200 pg/ml.

**Statistical analysis**

Student's t-test was used for the analysis of age and gender distribution between the case and the control groups. One-way ANOVA or t-test was used for the comparisons between the two groups using SPSS software (11.5, Chicago, Illinois). When the data points were less than 30 in categories, the normality of data was checked and parametric or non parametric (Kruskal-Wallis and Mann-Whitney) analyses were performed. Statis-tically significant differences were defined as comparisons resulting in p<0.05.

## Results

**Fig 1:**
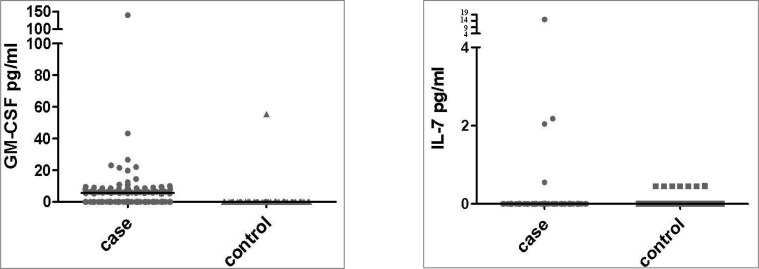
The comparison of the IL-7 and GM-CSF levels between patients and controls. The median values are shown by black lines in the graphics

A significantly higher level of GM-CSF (12.49±27.28 pg/ml) was found in the sera of patients with colorectal cancer compared with healthy age/sex matched controls (2.21 ± 15.68 pg/ml), (P= 0.013). Due to the high variance of data the analysis was performed by non-parametric comparison of the means (Mann-Whitney) and the results were confirmed (P=0.000). However, there was no significant difference between the level of IL-7 in sera of patients (0.16 ± 1.38 pg/ml) and healthy controls (0.06 ± 0.16 pg/ml) which was also confirmed by the non-parametric statistical evaluation (P=0.088). A high percentage of CRC patients (73 out of 124, 59%) had some level of GM-CSF in their sera while only one out of 50 (2%) healthy controls were found positive for GM-CSF (Roc curve cut off point= 5.04 pg/ml). Conversely, a higher number of healthy controls (7 out of 50, 14%, figure 1) had some level of IL-7 in their sera compared to the CRC patients (4 out of 124, 3.23%, figure 1).

**Table 2:** The age and gender distribution among patients and controls

		Age		Gender		Total
		Age < 40	Age ≥ 40	Male	Female	
Case	patient	25	102	68	59	127
	normal	7	43	27	23	50
Total		32	145	95	82	177
P value		0.376		0.965		

We observed a significant difference (P=0.024) in the serum level of GM-CSF in patients with different histological grades of colorectal cancer. The level of GM-CSF showed an increasing trend with the loss of differentiation in the tumor. Conversely, the highest level of IL-7 was detected in patients with well differentiated tumors and no IL-7 was detected in patients with poorly differentiated tumors (Table 3).

A significant difference in the level of GM-CSF in sera of CRC patients with or without lymphatic invasion was also observed (P= 0.01). Accordingly, the level of GM-CSF was much higher in sera of patients with lymphatic invasion compared to those without lymphatic invasion (Table 4). No difference was observed in the level of IL-7 between the two groups of CRC patients.

**Fig 2:**
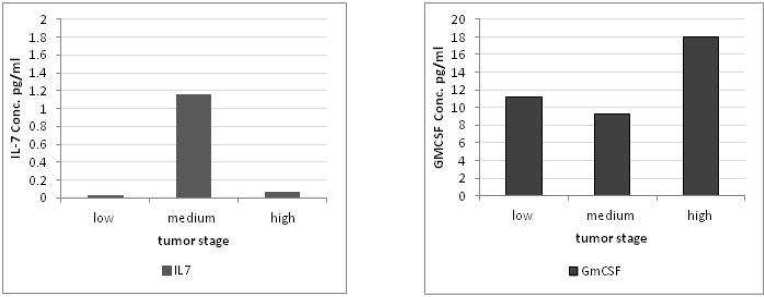
Levels of IL-7 and GM-CSF in patients with different stages of tumors. An increase in the level of IL-7 in the medium stage of colorectal tumors was accompanied by a decrease in the level of GM-CSF

**Table 3:** Well differentiated colorectal tumors induced more IL-7 production in the patients

Histological grade	N	IL-7 Mean ± SD	GM-CSF Mean ± SD
Well differentiated	86	0.20 ±1.65	10.14 ± 10.80
Moderately differentiated	17	0.16 ± 0.52	12.10 ± 20.39
Poorly differentiated	11	0	33.94 ± 81.83
P-value	114	0.911	0.024*

* Statistical significance when compared to histological grade.

We also compared the level of the two cytokines between CRC patients presenting low stages (Stages 1 and 2), medium stages (Stages 3a and 3b) and high stages (stages 3c and 4) tumors. We found a significant increase in the level of IL-7 in moderate stage tumors (P<0.05). This increase was concomitant with a decrease in the level of GM-CSF in sera of the patients with medium stage tumors (Figure 2).

The mean serum levels of GM-CSF did not show any difference between male (9.49 ± 8.80 pg/ml) and female (15.81 ± 38.36 pg/ml) CRC patients. There was also no significant difference between the level of IL-7 in male (0.04 ± 0.28 pg/ml) and female (0.29 ± 1.98 pg/ml) patients.

There was no significant correlation between tumor type, tumor side, tumor size, perineural invasion, vascular invasion and lymph node involvement with either of GM-CSF or IL-7 cytokines levels.

## Discussion

In this study we observed a significantly higher level of GM-CSF in Iranian patients with CRC compared to their age/sex matched controls. Previous studies have shown that the increased level of local cytokines in the site of tumor stimulates the immune system against tumor and CSFs have the most important role in this event (3). On the other hand, cytokines and growth factors produced by cancer cells or the stroma of tumor stimulate tumor growth and invasiveness (10). The detection of CSF receptors in several solid tumor cells including CRC cells may contribute to the stimulation of tumor by these cytokines (9). In a few investigations in other populations the level of GM-CSF in sera of CRC patients has been quite variable. One study has shown that elevated level of GM-CSF in sera of CRC patients does not correlate with prognosis or clinical features of CRC (5). A study found no GM-CSF in sera of CRC patients (25), while another group found a slight elevation of GM-CSF level in sera of CRC patients (26). It is shown that CRC cells not only express GM-CSF receptor but also secrete GM-CSF thereby stimulating their own proliferation (3). The odd, however, is that combining GM-CSF with chemotherapy leads to better treatment of many solid tumors with a mild inhibition of angiogenesis or moderate apoptosis in CRC cells (27-29). Moreover, colon cancer cells genetically engineered to secrete GM-CSF, have been shown to afford specific and long-lasting anti-tumor immunity (6).

The exact explanation for the variable results on the production of GM-CSF in CRC and the mechanisms behind its dual (or multiple) role against tumor cells is not known. However, it has been suggested that a slight elevation in the serum GM-CSF levels may reflect a protective response in cancer patients, while higher levels of autologous GM-CSF may stimulate the metastatic properties of cancer cells (26). Accordingly, the addition of recombinant GM-CSF at doses ranging between 30 pg/ml and 30 ng/ml did not appear to affect the poliferation of colorectal cancer cell lines in culture (22).

**Table 4:** Higher levels of GM-CSF in sera of colorectal cancer patients with lymphatic invasion compared to those without lymphatic invasion

		N	IL-7 [Mean ± SD]	GM-CSF [Mean ± SD]
Lymphatic invasion	Is seen	26	0.08 ± 0.43	24.49 ± 53.52
	Not seen	98	0.18 ± 1.54	9.31 ± 12.52
P-value		124	P= 0.752	P= 0.01^*^

A previous report showed that the level of IL-8 but not GM-CSF was significantly different between the well differentiated adenocarcinomas and other types of colorectal tumors (26), however, we found a significant correlation between the level of GM-CSF and the histological grade of the tumors. The poorly differentiated tumors were associated with an increase of the level of GM-CSF in the sera of patients. This was accompanied by the correlation of lymphatic invasion of the tumor and the level of GM-CSF. 

In our study, IL-7 was only elevated in 4 CRC patients and did not differ between patients and controls. Despite the low number of IL-7 positive CRC patients, an inverse pattern of GM-CSF and IL-7 increase was observed in the four IL-7 positive cases. Increase of IL-7 was accompanied by a decrease of the GM-CSF in those patients. The low number of IL-7 positive cases hampers to draw any conclusion but it may be suggestive of a beneficial role of IL-7 in CRC. It can also be a part of efforts of the immune system to respond to the tumor. There is limited information on the level of IL-7 in colorectal cancer and the results of the available studies are contradictory. 

In one study, IL-7 was only detectable in higher stages of the tumor. In our hands, IL-7 was only produced by tumors in stage 3a where the production of GM-CSF decreased. On the contrary, there are other reports showing that IL-7 is increased in colorectal cancer and it can be used as a diagnostic or prognostic factor in this cancer (18- 20). Moreover, the transition from stage 3 to stage 4 of colorectal cancer is characterized by an increase of the serum level of IL-7 (18- 20).

The mean concentrations of both IL-7 and GM-CSF are reported to be high in females with colorectal cancer but did not reach the significant level (10). Mroczko et al. observed higher serum levels of IL-3, GM-CSF and M-CSF in males in comparison to females, but again, these differences were not significant (10). Our results, however, did not show any difference between the levels of GM-CSF and IL-7 in male and female patients.

Currently it is difficult to interpret the discrepancy of the IL-7 data obtained in our study and some previous studies but as mentioned by others, what actually operate in vivo are the cytokine cascades and cytokine networks (26). In addition, part of these differences may be due to the diversity of the cells of a specific tumor type in different patients. Moreover, as shown for breast tumor cells, even in the same host, tumor cells show transcriptional diversity (30). Therefore, obtaining a consensus pattern of cytokine production by colorectal (and other) tumor cells in different individuals needs the evaluation of a broader range of cytokines in a greater number of patients.

We did not find any correlation between tumor type, tumor side, tumor size, perineural invasion, vascular invasion, and lymph node involvement with either GM-CSF or IL-7 cytokines. Similarly, another study reported no correlation between the serum levels of GM-CSF and the clinicopathological findings in colorectal

cancer (26).

In summary, due to a high percentage of patients with elevated serum level of GM-CSF, we suggest that GM-CSF can be used as a tumor marker in Iranian colorectal cancer patients, however, the specificity and sensitivity of the test should be defined in future studies. We also suggest that the role of IL-7 in colorectal cancer needs more investigation and may provide a new target in the immunotherapy of colorectal cancer.
